# Data of REEs (Ce, Nd, Th) analysis from Bangka tin tailing applying froth flotation method using sodium oleate and KClO_3_

**DOI:** 10.1016/j.dib.2024.111157

**Published:** 2024-11-23

**Authors:** Wiwik Dahan, Djoko Hartanto, Ratna Ediati, Rita Sundari, Subandrio, Irfan Marwanza

**Affiliations:** a Department of Mining Engineering, Faculty of Earth Technology and Energy, University of Trisakti, Jakarta 11450, Indonesia; b Department of Chemistry, Institute of Teknologi Sepuluh Nopember, Surabaya 60111, Indonesia; c Department of Mechanical Engineering, University of Mercu Buana, Jakarta 11650, Indonesia

**Keywords:** Collector, Flotation agent, Mining exploitation, Tailing, XRD, XRF

## Abstract

This article presented the data of REEs (Rare Earth Elements) analysis from exploitation of Bangka tin tailing, Indonesia. Nowadays, REEs have broad applications in modern industry such as computer memory, DVDs, rechargeable batteries, cell phones, catalytic converters, fluorescent lighting, negative ion generators, and much more. A 30 min. and 400 rpm froth flotation method has utilized 0.06 M sodium oleate flotation agent, 0.07 M KClO_3_ depressant, and 2.0 M HCl for pH arrangement of 10.0 g sample to analyse REEs from 170 mesh Bangka tin tailing at 25 °C. The analysis is found to be cerium (1.60 %, pH 7.0), neodymium (0.70 %, pH 7.0), and thorium (0.95 %, pH 8.0) in the collector, while at the same time, the concentrations of cerium (9.45 %, pH 7.0), neodymium (3.15 % pH 7.0), and thorium (3.90 %, pH 8.0) in the tailing (depressant) applying froth flotation method. Based on the variation of KClO_3_ concentrations at the given condition (0.06 M sodium oleate, pH 7.0, 30 min., 400 rpm. 25 °C), the recovery of REEs in the collector using froth flotation method is as follows: the highest concentrations of cerium (11.80 %) and neodymium (3.93 %) were obtained at 0.005 M KClO_3_ and pH 7.0, while the highest concentrations of thorium (4.50 %) obtained at KClO_3_ free (none KClO_3_) at the same pH. The utilization of sodium oleate flotation agent and KClO_3_ depressant in the flotation REE recovery from tin tailing can be viewed as a new contribution of this study since unpublished previous work applied palmitate collector and no any depressant for REE recovery from the same mining area. The contribution of REE analysis from Bangka tin tailing applying froth flotation method is valuable for mining industry.

Specifications Table

**Table:** 

Subject	Mining engineering
Specific subject area	Mineral analysis
Type of data	Tables and Graphs
Data collection	Mineral sample was exploited from tin mining tailing in Bangka island, Indonesia. The mineral sample was crushed and screened to obtained particle size of 170 mesh. The REEs from mineral sample were determined by XRF (X-Ray Fluorescence) applying froth flotation method. XRF device was applied to determine concentration of elements and their oxides. XRD (X-Ray Diffraction) device was applied to examine minerals containing REEs.
Data source location	Department of Mining Engineering, Faculty of Earth Technology and Energy, University of Trisakti, Jakarta 11,450, Indonesia, is the owner of the data presented in this article. The experimental site is located at 2°15′S latitude and 106°00′E longitude with an altitude of 100 m above sea level (Fig. 1).
Data accessibility	Repository name: Mendeley Data https://data.mendeley.com/datasets/wbgsbcn6zd/1 and https://data.mendeley.com/datasets/6j2v35ykky/1 Data identification number: 10.17632/wbgsbcn6zd.1 and 10.17632/6j2v35ykky.1 Direct URL to data: 10.17632/wbgsbcn6zd.1

## 1 Value of the Data

• Data can verify the existence of precious REEs in Bangka tin tailing, Indonesia, which is really worth for further minerals exploitation at the same area.
• Data provide estimation of concentrations of REEs with respect to Ce (cerium), Nd (neodymium), and Th (thorium) in Bangka tin tailing, Indonesia, as a result of using froth flotation method.
• Data can be reused for future research in other minerals exploration related to separation of REEs from mining tailing applying another routes compared to froth flotation method.
• Mining and chemical engineers can take benefit from this dataset to reveal mining exploration for REEs production to be utilized in modern industry.
• Policymakers can apply the dataset to consider decisions related to financial source, platform of research initiative, and regional mitigation. It informs the understanding of importance and impact related to mining research in the corridor of REE recovery.

## 2 Background

The data of REEs existence in Bangka tin tailing, Indonesia, is very important for the information of precious mineral content in tin tailing. Bangka Island in Indonesia that is very substantial for tin production where it produces major tin concentrate and minor tin tailing containing valuable REEs including lanthanum, cerium, neodymium, thorium, uranium, ytterbium, and more. Nowadays, REE is very remarkable in the development of modern industry [[1], [2], [3]]. Froth flotation method is commonly used for separation and recovery of REE minerals [[4], [5], [6]]. In fact, flotation method has been broadly applied in many separation processes related to ore gangue minerals. Shihao Ding et al. [7] reported the mechanism of bubble particle detachment in flotation method addressing to tangential and vertical directions and found that vertical direction was more stable. However, this study has emphasized on REE recovery from ore gangue mineral and therefore, the extensive discussion of flotation method is beyond the scope of this study. This work has applied froth flotation method using sodium oleate and KClO_3_ attempted to concentrate the REEs in collector based on hydrophobic oleate and hydrophilic KClO_3_ characters. In relation to this study using sodium oleate collector for flotation monazite recovery from kaolinite, Abaka-Wood et al. [8] discussed the concept of mineral and collector interaction and found significant interaction between monazite and sodium oleate yielding increased monazite recovery in flotation method. It should be noted that REE is a part of monazite minerals. In earlier year, Abaka-Wood et al. [9] applied sodium oleate collector with starch and sodium silicate depressants and found selective flotation of REE oxides from hematite-quartz mixture due to starch and sodium silicate depressants. This study used tin tailing as raw material instead of kaolinite or mixture of hematite and quartz. On other occasion, Abaka-Wood et al. [10] reported sodium oleate and hydroxamic acid collector attract more REE while sodium silicate and starch depressants attract more iron oxide and silicate tailing in flotation of REE. For measurements, this study applied XRF (X-ray Fluorescence) to determine elements in mining minerals and XRD (X-ray Diffraction) to observe the existence of REE minerals.

## 3 Data Description

**Fig. 1:**
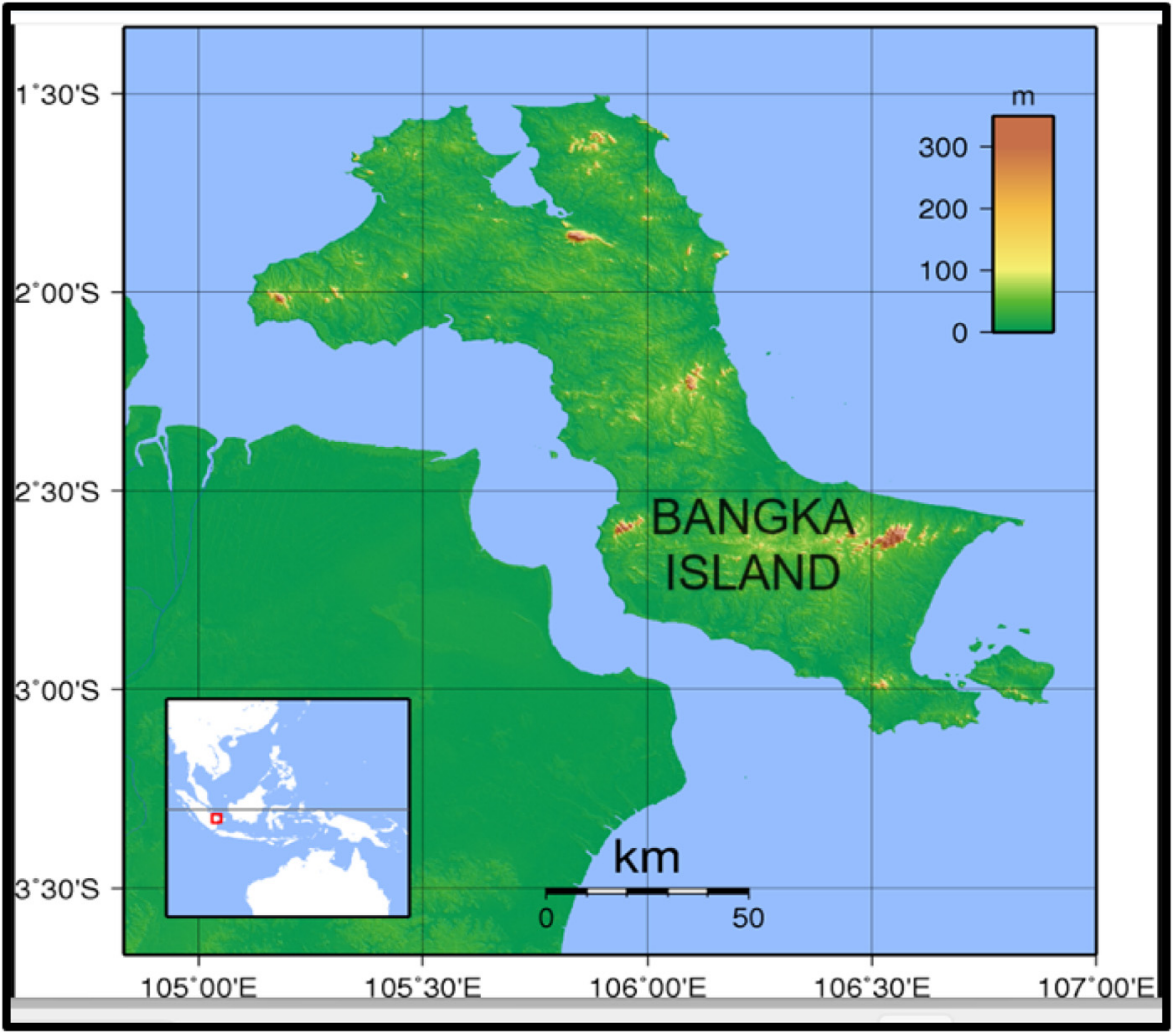
Map location of Bangka Tin Mining, Indonesia.

(i) The mineral sampling was taken in the middle area of Bangka Island, Indonesia, as shown in Fig. 1. Tables 1 and 4 present the dataset of REEs obtained from XRF measurement as concentrations of elements and their oxides yielded by froth flotation method applying sodium oleate and KClO_3_ represented as collectors and tailings.

(open pit mining).

**Table 1:** Data of elements in Bangka Tin Tailing, Indonesia (effect of pH).

element	Initial sample from Bangka Tin Tailing									
	Initial sample (%)	Initial sample revision	pH 6.0		pH 7.0		pH 8.0		pH 9.0	
			Collector (%)	Tailing (%)	Collector (%)	Tailing (%)	Collector (%)	Tailing (%)	Collector (%)	Tailing (%)
Zr	54.30	54.60	8.50	60.90	42.00	60.20	10.90	59.50	1.70	59.90
Ce	10.10	9.44	1.40	9.75	1.60	9.45	1.30	10.10	0.00	10.80
Fe	7.53	8.38	4.59	5.60	37.70	6.57	5.17	5.91	25.40	5.85
Sn	4.29	3.60	0.00	4.69	0.00	4.78	0.00	4.92	0.00	4.77
Ti	4.03	3.76	0.46	2.84	0.00	3.52	0.51	3.04	0.00	2.80
Th	3.40	3.30	0.87	3.60	0.00	3.60	0.95	3.90	0.00	4.00
Si	3.40	3.70	0.00	2.10	0.00	2.20	0.00	2.10	0.00	2.10
S	3.21	3.28	2.58	0.00	0.00	0.00	2.29	0.00	2.10	0.06
Nd	3.20	3.02	0.47	3.12	0.70	3.15	0.51	3.21	0.00	3.47
P	2.10	2.00	0.99	2.00	3.20	2.00	1.00	2.10	0.86	2.20
Y	1.40	1.30	0.39	1.50	0.00	1.50	0.41	1.60	0.00	1.40
Hf	1.21	1.23	0.00	1.11	0.00	1.13	0.00	1.13	0.00	1.15
Pb	0.87	0.85	0.37	0.76	2.70	0.96	0.00	0.83	0.40	0.81
As	0.40	0.41	0.14	0.32	0.65	0.42	0.17	0.37	0.08	0.35
Ca	0.26	0.28	1.20	0.80	6.28	0.35	1.00	0.27	1.00	0.39
W	0.09	0.08	0.00	0.00	0.00	0.11	0.00	0.00	0.00	0.00
Zn	0.03	0.00	0.03	0.00	0.40	0.00	0.07	0.00	0.09	0.00
Cl			77.70	0.00	0.00	0.00	74.90	0.00	67.40	0.00
Ni			4.00	0.00	0.37	0.00	0.00	0.00	0.07	0.01
Cu			14.00	0.00	1.50	0.00	0.17	0.00	0.20	0.00

Noted: 1) Yellow colour denoted data of REE (cerium, thorium, and neodymium).

2) Effect of pH in froth flotation method (0.06 M sodium oleate, 0.07 M KClO_3_).

3) Result data of XRF measurement.
(ii) Table 1, Table 2 reveal data of elements and related element oxides in Bangka tin tailing at varied pH (pH 6.0– 9.0). Table 1, Table 2 used 0.06 M sodium oleate, 0.07 M KClO_3_, and 2.0 M HCl. Both Table 1, Table 2 show very low concentrations of thorium and thorium oxide (< 1.00 %) in collectors at given varied pH. The fluctuated concentrations of REE (Ce, Nd, and Th) in collectors at varied pH are affected by acid hydrolysis and interaction between REE and sodium oleate flotation agent.

**Table 2:** Data of minerals (element oxides) in Bangka Tin Tailing, Indonesia (effect of pH).

mineral	Initial sample from Bangka Tin Tailing									
	Initial sample (%)	Initial sample revision	pH 6.0		pH 7.0		pH 8.0		pH 9.0	
			Collector (%)	Tailing (%)	Collector (%)	Tailing (%)	Collector (%)	Tailing (%)	Collector (%)	Tailing (%)
ZrO_2_	50.20	50.10	10.00	59.10	40.00	58.40	12.70	57.60	1.90	58.20
CeO_2_	8.84	8.20	1.60	8.87	1.50	8.58	1.40	9.19	0.00	9.81
Fe_2_O_3_	7.59	8.40	5.76	5.88	38.70	6.90	6.47	6.21	30.00	6.17
SnO_2_	3.91	3.30	0.00	4.45	0.00	4.53	0.00	4.66	0.00	4.53
TiO_2_	4.79	4.45	0.68	3.52	0.00	4.35	0.75	3.76	0.00	3.47
ThO_2_	2.70	2.60	0.87	3.00	0.00	3.00	0.94	3.20	0.00	3.30
SiO_2_	5.90	6.40	0.00	3.80	0.00	3.90	0.00	3.70	0.00	3.60
SO_3_	5.80	5.97	5.91	0.00	0.00	0.00	5.21	0.00	4.70	0.10
Nd_2_O_3_	2.64	2.47	0.48	2.69	0.60	2.70	0.52	2.76	0.00	2.99
P_2_O_5_	3.90	3.60	2.10	3.70	5.80	3.70	2.20	3.80	1.80	4.00
Y_2_O_3_	1.20	1.20	0.43	1.30	0.00	0.00	0.45	0.00	0.00	1.30
HfO_2_	1.00	1.01	0.00	0.96	0.00	0.98	0.00	0.98	0.00	1.00
PbO	0.66	0.64	0.35	0.60	2.10	0.75	0.45	0.65	0.40	0.64
As_2_O_3_	0.37	0.38	0.16	0.31	0.61	0.40	0.19	0.35	0.06	0.34
CaO	0.26	0.28	1.40	0.83	6.46	0.37	1.30	0.28	1.20	0.41
WO_3_	0.08	0.07	0.00	0.00	0.00	0.10	0.00	0.00	0.00	0.00
ZnO	0.02	0.00	0.03	0.00	0.30	0.00	0.08	0.00	0.09	0.00
Cl			69.90	0.00	0.00	0.00	67.10	0.00	58.80	0.00
NiO			0.04	0.00	0.33	0.00	0.00	0.00	0.08	0.00
CuO			0.16	0.00	1.40	0.00	0.19	0.00	0.21	0.00

Noted: 1) Yellow colour denoted data for oxides of cerium, thorium, and neodymium.

2) Effect of pH in froth flotation method (0.06 M sodium oleate, 0.07 M KClO_3_).

3) Result data of XRF measurement.
(iii) Table 3, Table 4 reveal XRF data for REE elements and related oxides applying varied KClO_3_ concentrations (from none to 0.05 M KClO_3_) using 0.06 M sodium oleate at pH 7.0. Table 3 reveals the highest concentrations of Ce (11.80 %) and Nd (3.93 %) obtained in the collector at 0.005 M KClO_3_, however, the highest concentration of Th (4.50 %) obtained in the collector in the absence of KClO_3_, all at the condition of pH 7.0 and using 0.06 M sodium oleate. Table 4 related to element oxides shows the same trend as shown by XRF data of Table 3 with respect to Ce, Nd, and Th.

**Table 3:** Data of elements in Bangka Tin Tailing, Indonesia (effect of varied KClO_3_).

Element	From Original Bangka Tin Tailing									
	none KClO_3_		0.005 M KClO_3_		0.01 M KClO_3_		0.03 M KClO_3_		0.05 M KClO_3_	
	Collector (%)	Tailing (%)	Collector (%)	Tailing (%)	Collector (%)	Tailing (%)	Collector (%)	Tailing (%)	Collector (%)	Tailing (%)
Si	0.61	2.30	2.00	2.50	0.44	3.70	0.56	2.20	0.44	2.10
P	0.98	2.70	1.70	2.90	1.40	2.50	2.10	2.30	1.40	2.10
Ca	0.78	0.42	0.39	0.65	1.50	0.27	2.31	0.31	0.64	0.38
Ti	1.91	3.09	2.13	4.46	1.55	4.49	1.41	2.90	1.14	3.30
Fe	15.70	5.74	5.17	8.31	11.40	8.26	9.61	4.66	6.54	6.05
As	0.57	0.34	0.23	0.57	0.45	0.49	0.45	0.33	0.23	0.39
Y	1.60	1.70	1.50	1.70	1.50	1.50	1.80	1.60	0.75	1.60
Zr	41.60	54.20	39.70	47.90	37.70	52.70	32.30	57.00	22.00	58.60
Sn	2.10	6.14	6.35	5.40	3.47	4.43	2.88	6.30	0.77	5.06
Ce	6.57	13.00	11.80	13.20	4.05	11.00	5.60	12.10	3.66	10.80
Nd	2.10	4.20	3.93	4.44	1.50	3.58	2.10	3.98	1.30	3.47
Hf	1.10	1.17	1.19	1.20	0.93	1.20	1.00	1.15	0.65	1.12
Pb	2.70	0.72	0.59	1.30	0.98	1.10	1.30	0.77	0.83	0.89
Th	4.50	4.30	3.90	4.80	3.40	3.80	3.80	4.20	1.70	3.90
S	0.00			0.50				0.10	2.50	0.05
Ni	0.04		0.01	0.01	0.04		0.06		0.02	0.01
Zn	0.08				0.07				0.04	
Yb	0.06		0.07	0.20	0.10		0.20		0.06	0.10
*Re*	0.07				0.08				0.03	

Noted: 1) Yellow colour denoted data of REE (cerium, thorium, and neodymium).

2) Effect of varied KClO_3_ in froth flotation method at pH 7.0 (0.06 M sodium oleate, 2.0 M HCl).

3) Result data of XRF measurement.

**Table 4:** Data of minerals (element oxides) in Bangka Tin Tailing, Indonesia (effect of varied KClO_3_).

Mineral	From Original Bangka Tin Tailing									
	none KClO_3_		0.005 M KClO_3_		0.01 M KClO_3_		0.03 M KClO_3_		0.05 M KClO_3_	
	Collector (%)	Tailing (%)	Collector (%)	Tailing (%)	Collector (%)	Tailing (%)	Collector (%)	Tailing (%)	Collector (%)	Tailing (%)
SiO_2_	1.10	4.00	3.40	4.40	0.80	6.50	2.30	3.80	0.86	3.80
P_2_O_5_	1.80	4.90	3.20	5.30	2.60	4.50	4.00	4.30	2.80	3.90
CaO	0.82	0.44	0.40	0.67	1.60	0.28	2.44	0.32	0.75	0.39
TiO_2_	2.70	3.82	2.63	5.46	1.90	5.45	1.77	3.59	1.58	4.09
Fe_2_O_3_	16.70	6.02	5.44	8.62	11.90	8.51	10.20	4.91	7.74	6.37
As_2_O_3_	0.56	0.33	0.22	0.55	0.43	0.46	0.44	0.32	0.25	0.37
Y_2_O_3_	1.50	1.60	1.40	1.50	1.30	1.40	1.70	1.50	0.78	1.50
ZrO_2_	45.9	52.50	51.00	45.80	46.60	49.90	35.20	55.40	24.10	56.90
SnO_2_	2.00	5.82	6.03	5.10	3.36	4.13	2.69	5.99	0.82	4.80
CeO_2_	6.08	11.80	10.70	11.90	3.66	9.79	5.15	11.00	3.74	9.79
Nd_2_O_3_	1.90	3.61	3.38	3.76	1.30	3.01	1.80	3.43	1.20	2.98
HfO_2_	1.00	1.01	1.03	1.00	0.80	1.02	0.90	1.00	0.63	0.97
PbO	2.10	0.56	0.46	1.00	0.77	0.81	1.00	0.60	0.73	0.70
ThO_2_	3.80	3.60	3.20	3.90	2.80	3.10	3.20	3.50	1.60	3.20
SO_3_	0.10			0.90				0.20	5.40	0.10
NiO	0.03		0.01	0.01	0.04		0.06		0.02	0.01
ZnO	0.08				0.06				0.04	
Yb_2_O_3_	0.05		0.06	0.10	0.10		0.10		0.06	0.08
Re_2_O_7_	0.07				0.08				0.03	

Noted: 1) Yellow colour denoted data for oxides of cerium, thorium, and neodymium.

**Fig. 2:**
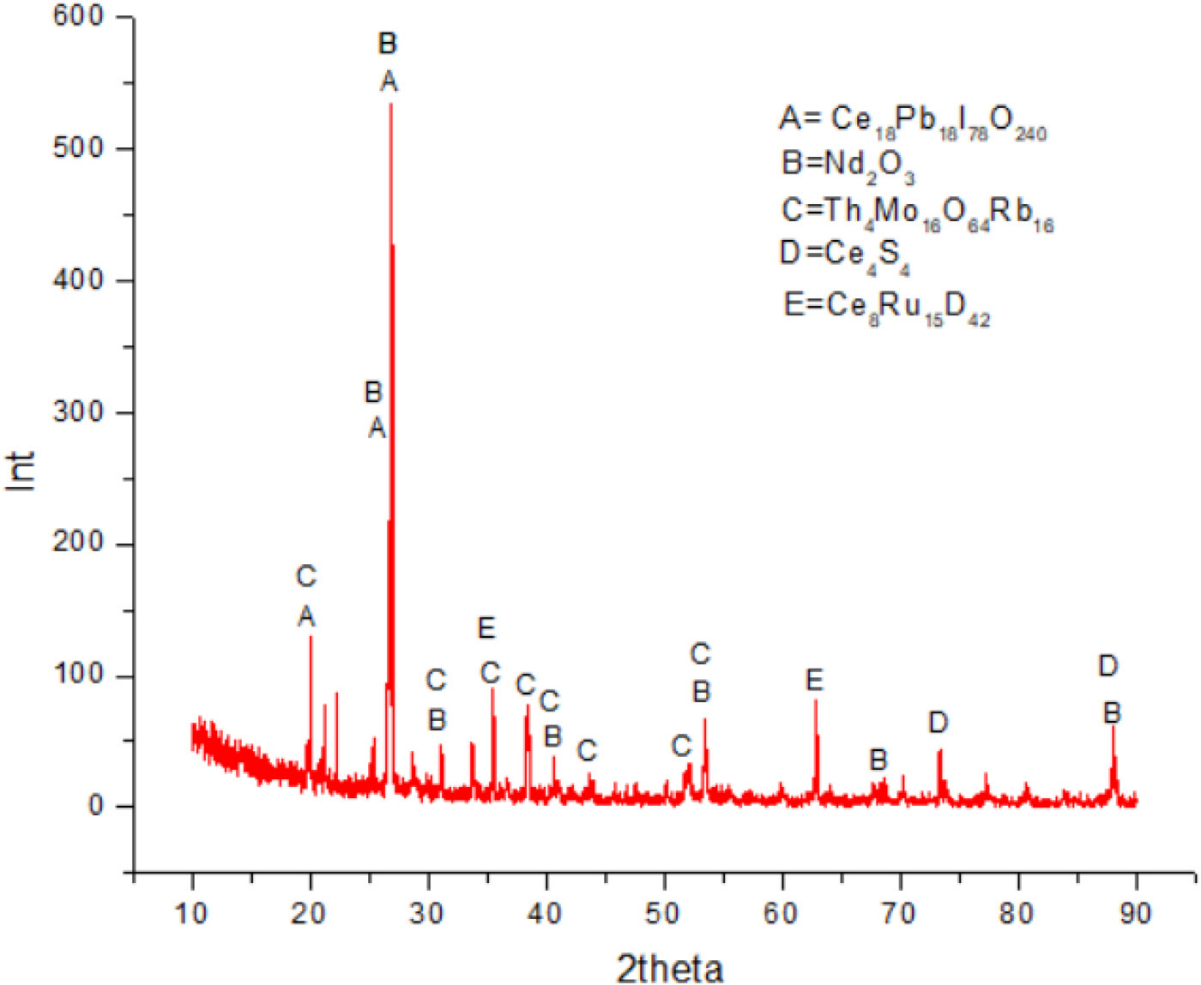
XRD graph of initial mineral sample of Bangka tin tailing.

**Fig. 3:**
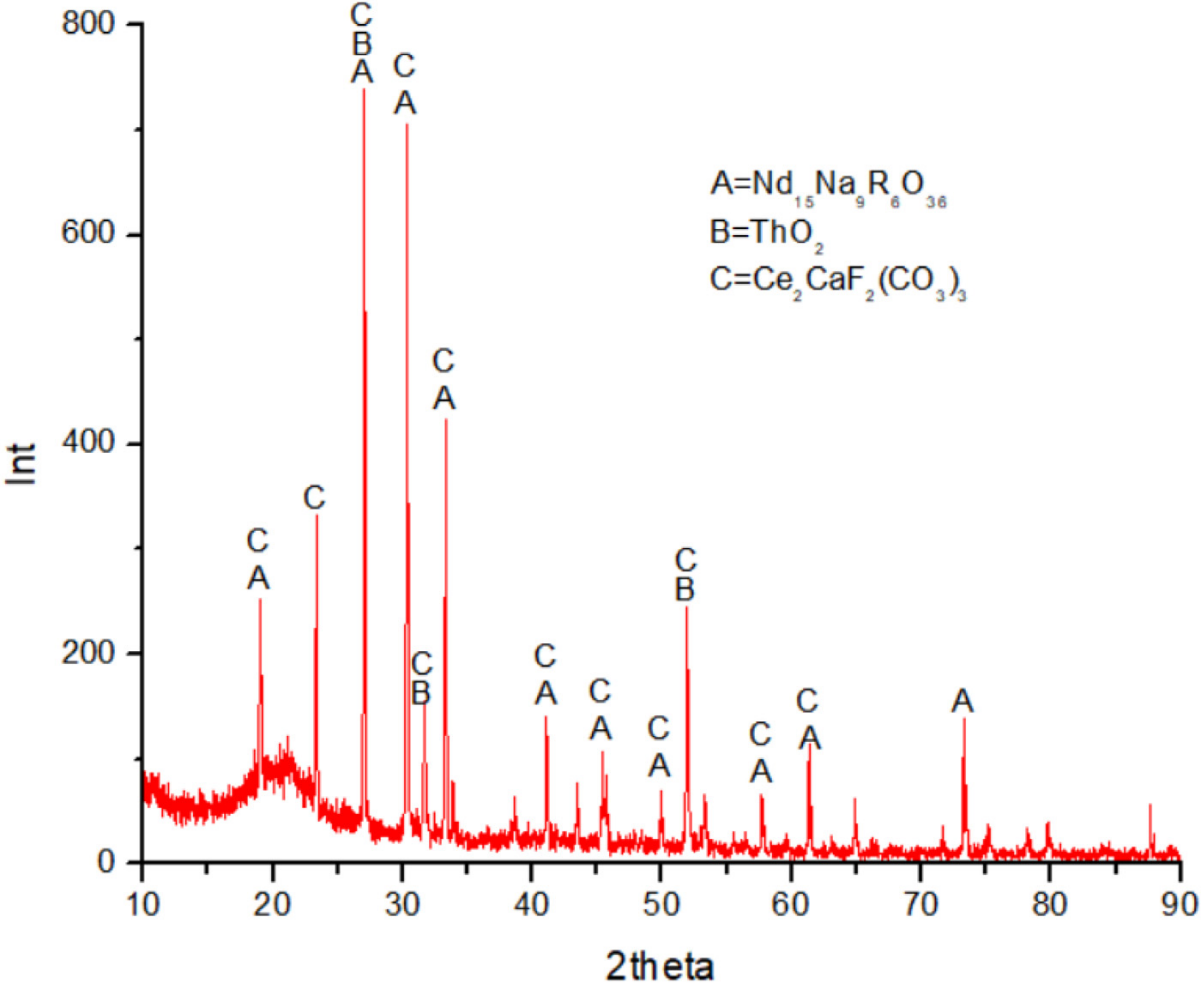
XRD graph of mineral sample in collector froth flotation method at pH 6.0. Sodium oleat 0.06 M KClO_3_ 0.07 M.
(vi) Fig. 4, Fig. 5 show the concentrations of Ce and Nd minerals decreased at pH 7 and 8, respectively. At pH 9.0 the Ce, Nd, and Th minerals were totally disappeared in the collector and therefore, the graphs of XRD at pH 9.0 not represented.

2) Effect of varied KClO_3_ in froth flotation method at pH 7.0 (0.06 M sodium oleate, 2.0 M HCl).

3) Result data of XRF measurement.
(iv) Furthermore, Table 1, Table 2 reveal the concentrations of thorium and thorium oxide that are not detected using 0.07 M KClO_3_ at pH 7.0, however, Table 3 shows the concentration of thorium found to be 1.70 % and Table 4 shows 1.60 % thorium oxide using 0.05 M KClO_3_ at pH 7.0. KClO_3_ inorganic salt has polar or hydrophilic character; it implies that less KClO_3_ yielding more REE in the collector.
(v) Fig. 2 shows the XRD graph of initial sample before froth flotation represented sharp peaks of REE (Ce, Nd, and Th) minerals. Fig. 3 shows the XRD graph of mineral sample in collector of flotation cell at pH 6.0, which Th mineral is not detectable due to very low Th mineral concentration.

**Fig. 4:**
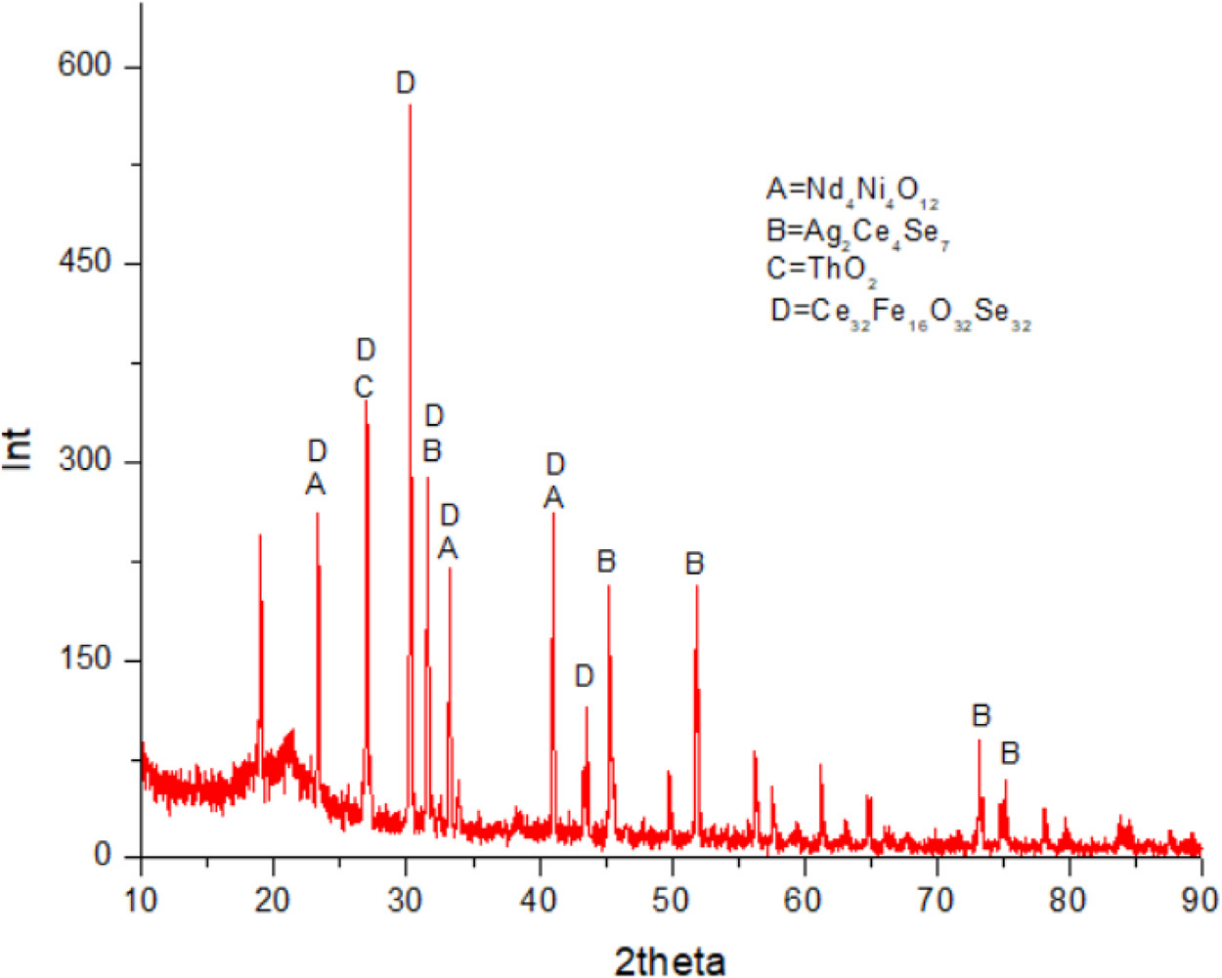
XRD graph of mineral sample in collector froth flotation method at pH 7.0. Sodium oleat 0.06 M KClO_3_ 0.07 M.

**Fig. 5:**
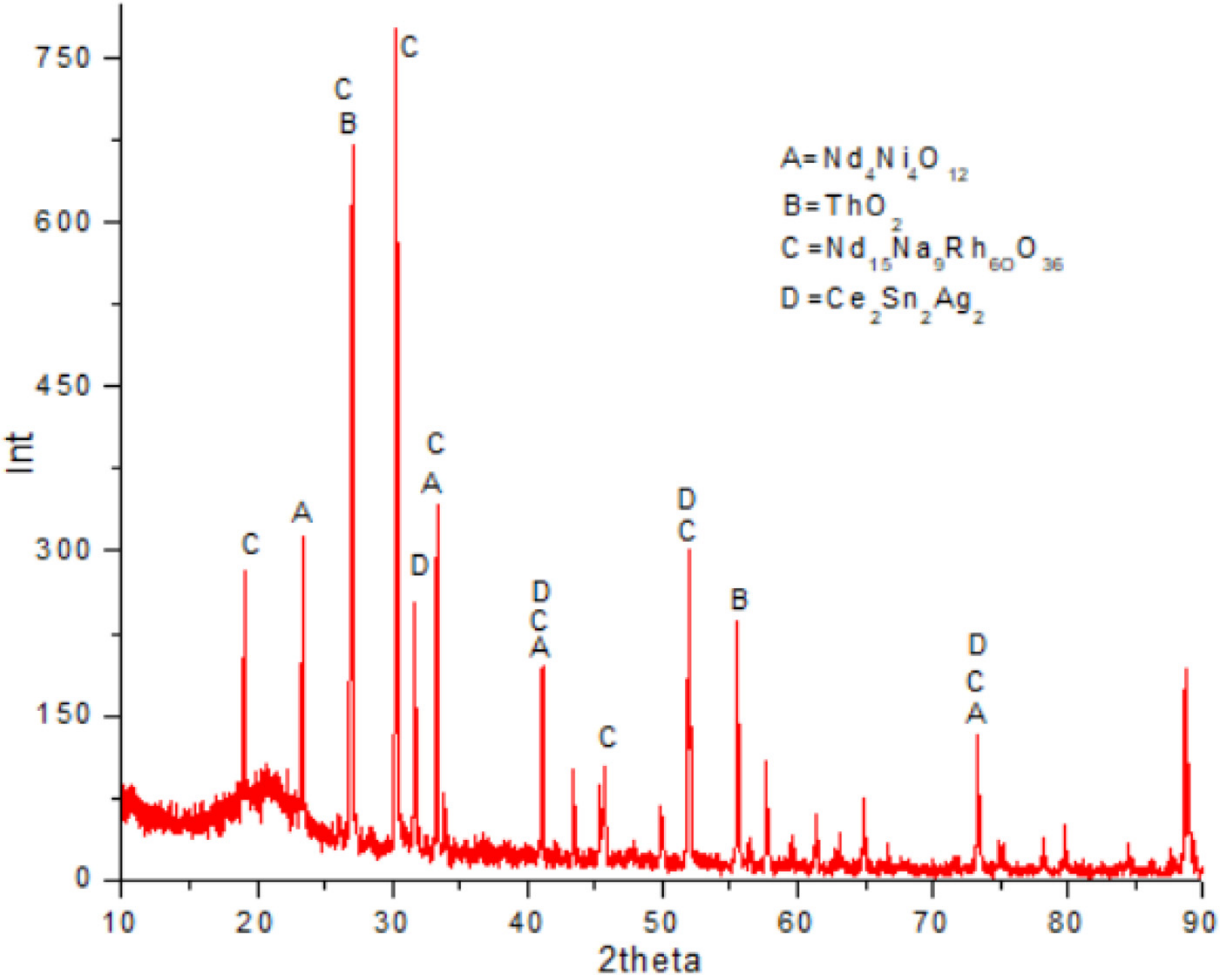
XRD graph of mineral sample in collector froth flotation method at pH 8.0. Sodium oleat 0.06 M KClO_3_ 0.07 M.
(vii) Fig. 6 represents all Fig. 2, Fig. 3, Fig. 4, Fig. 5 including XRD graph of initial mineral sample and mineral samples in collectors of flotation cell consecutively at pH 6, 7, and 8. Fig. 6 shows the change of concentrations of Ce, Nd, and Th minerals affected by type of element, REE bonding in minerals influenced by medium acidity causing some REE minerals attracted to tailing in flotation cell.

**Fig. 6:**
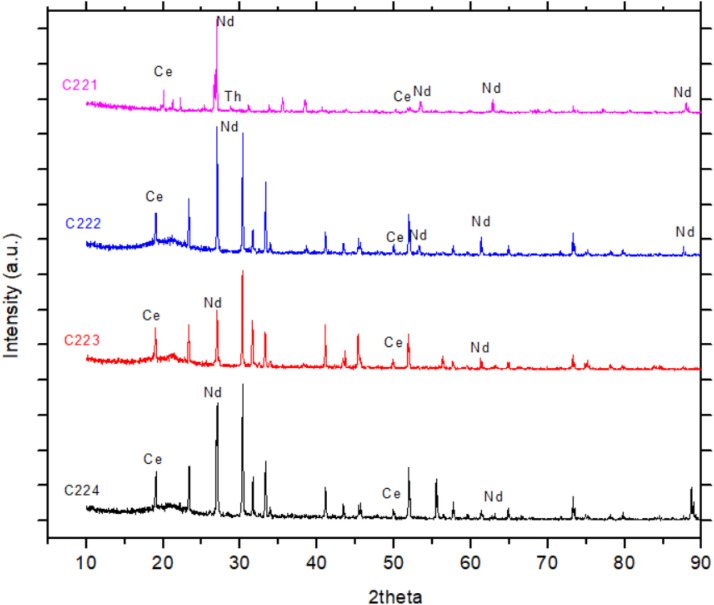
XRD graphs of mineral samples. C221 = initial sample. C222 = collector pH 6.0. C223 = collector pH 7.0. C224 = collector pH 8.0 (note: Ce, Nd, and Th existed with other elements in minerals).

## 4 Experimental Design, Materials and Methods

### 4.1 Experimental steps

The current work is described by the following steps:

• The mineral sample was taken from open pit mining in Bangla Island, Indonesia (as shown in Fig. 1).
• The mineral sample was crushed and screened until 170 mesh sample size obtained.
• The 10.0 g of 170 mesh mineral sample was put in a 200 ml container for flotation process. The flotation was conducted at 25 °C, 30 min., and 400 rpm addressing to previous work related to monazite recovery [11]. The flotation applied sodium oleate as floating agent, KClO_3_ as depressant, and HCl as pH adjuster.
• The froth flotation processes were varied at pH 6 – 9 and KClO_3_ concentrations (none KClO_3_, 0.005 M, 0.01 M, 0.03 M, and 0.05 M).
• The order of material addition in flotation method can be summarized as follows:
  1. 10.0 g tin tailing was added into flotation cell.
  2. 3.0 g sodium oleate flotation agent (frother) was added into flotation cell.
  3. 180 ml distilled water was added.
  4. The mixture was stirred at 400 rpm for 10 min.
  5. HCl was added to arrange the given solution pH.
  6. The mixture was conditioned for 10 min.
  7. Aeration was conducted to yield foam.
• XRF measurements were conducted on collector and tailing of froth flotation process to obtain the dataset of elements and their oxides of mineral at varied pH and KClO_3_ concentrations.
• XRD examinations were conducted on collectors of froth flotation process at varied pH to represent XRD graphs.

### 4.2 Materials

The current work applied analytical grade chemicals, i.e. sodium oleate (Sigma Aldrich) as flotation agent, KClO_3_ (Scharlau) as depressant, and HCl (Merck) to arrange the pH of solutions. The solution of sodium oleate was made by dissolving 3.0 g sodium oleate in 180 ml distilled water, solution of KClO_3_ made by dissolving 1.5 g, and 37 % conc. HCl was equal to about 12 M and diluted with distilled water to a given concentration (2 M). The addition of HCl in the solution was used to control the varied pH. The concentration of KClO_3_ was adjusted to obtain the given concentrations (0.005 M, 0.01 M, 0.03 M, 0.05 M, and 0.07 M). The mineral sample was taken from tailing of tin open pit mining exploitation in Bangka Island, Indonesia, about 100 m above sea level.

### 4.3 Instrumentation

The principle of froth flotation method addressing to current work is to separate REE minerals into collector (concentrate) and tailing (depressant) based on differences in the ability of air bubbles related to selective adhered to specific REE mineral surface in aqueous solution. The current work applied a one litre flotation cell made of plexiglass with 45 mm diameter stainless steel vane running by 180 watt motor power and JW5622BI4 motor type able to load particle size of 0.5 mm (max.).

The XRF analysis was conducted by PANalytical/ Minipal 4 provided with 9 W X-ray tube Rh (0.1 mA and 20 kV), 5 tube filters, equipped with a high resolution Silicon Drift Detector and a 12-position sample tray with sample spinner. The XRF analysis applied Monazite Reference Material to validate the standard curve.

The XRD patterns were recorded by PANalyticalX'pert PRO XRD with CuKα incident radiation (1.54060 A), from a Cu anode operated at 40 kV and 30 mA. The XRD patterns of samples were measured in continuous scanning mode with a speed of 0.02°/min. in the scanning range 2θ from 10° to 90°. Silicon was used as the standard material.

## Limitations

Not applicable

## Ethics Statement

Authors have read and follow the ethical requirements for publication in Data in Brief and confirming that the current work does not involve human subjects, animal experiments, or any data collected from social media platforms.

## CRediT Author Statement

**Wiwik Dahani:** Conceptualization, Methodology, Software, Data curation; **Djoko Hartanto:** Writing – review & editing; **Ratna Ediati:** Writing – review & editing; **Rita Sundari:** Conceptualization, Supervision, Writing, Original draft preparation; **Subandrio:** Methodology, Software, Validation; **Irfan Marwanza:** Supervision, Visualization, Investigation.

## Data Availability

Mendeley DataXRF data using varied KClO3 (Original data). Mendeley DataDataXRF measurement effect pH (Original data). Mendeley DataDataXRF measurement effect KClO3 (Original data).
